# Endothelin mediates sex-differences in acclimation to high salt diet in rats

**DOI:** 10.1186/s13293-023-00555-2

**Published:** 2023-10-10

**Authors:** Victoria L. Nasci, Rawan N. Almutlaq, David M. Pollock, Eman Y. Gohar

**Affiliations:** 1 Division of Nephrology and Hypertension, Department of Medicine, Vanderbilt University Medical Center, Nashville, TN USA; 2 Division of Nephrology, Department of Medicine, University of Alabama at Birmingham, Birmingham, AL USA

**Keywords:** Endothelin receptor, Sex differences, Natriuresis

## Abstract

**Introduction:**

Current understanding of sodium (Na^+^) handling is based on studies done primarily in males. Contrary to the gradual increase in high salt (HS) induced natriuresis over 3–5 days in males, female Sprague Dawley (SD) rats have a robust natriuresis after 1 day of HS. Renal endothelin-1 (ET-1) signaling, through ET receptor A and B, is an important natriuretic pathway and was implicated in our previous dietary salt acclimation studies, however, the contribution of ET receptors to sex-differences in acclimation to dietary Na^+^ challenges has yet to be clarified. We hypothesized that ET receptors mediate the augmented natriuretic capacity of female rats in response to a HS diet.

**Methods:**

To test our hypothesis, male and female SD rats were implanted with telemeters and randomly assigned to treatment with A-182086, a dual ET_A_ and ET_B_ receptor antagonist, or control. 24-h urine samples were collected and assessed for electrolytes and ET-1. Studies were performed on a normal salt (NS, 0.3% NaCl) diet and after challenging rats with HS (4% NaCl) diet for 1 day.

**Results:**

We found that A-182086 increased blood pressure in male and female SD rats fed either diet. Importantly, A-182086 eliminated sex-differences in natriuresis on NS and HS. In particular, A-182086 promotes HS-induced natriuresis in male rats rather than attenuating the natriuretic capacity of females. Further, the sex-difference in urinary ET-1 excretion in NS-fed rats was eliminated by A-182086.

**Conclusion:**

In conclusion, ET receptors are crucial for mediating sex-difference in the natriuretic capacity primarily through their actions in male rats.

## Highlights

- Female rats excrete a greater amount of sodium than male rats on the first day of increasing the dietary Na^+^ intake.
- Dual antagonism of ET receptors A and B with A-182086 augmented sodium excretion in male rats eliminating the observed sex difference in HS evoked natriuresis.
- Greater levels of urinary ET-1 are evident in NS-fed female rats and A-182086 eliminated these sex differences in urinary ET-1 excretion in female rats.

## Plain language summary

Sodium balance is essential for the human body. Sodium retention in the body can cause an increase in blood pressure. Historical understanding of sodium balance is based on studies done mostly in male subjects. Recently, we showed that male and female rats acclimate to a high salt diet differently. Male rats take 3–5 days to increase sodium excretion while female rats increase sodium excretion after 1 day. Endothelin-1 which signals through two receptors, endothelin receptor subtype A and B, is important for controlling sodium excretion by the kidneys. There are known sex-differences in the ratio and function of endothelin receptors in the kidney. However, the role of endothelin receptors in salt handling during acclimation to increased salt intake is not clear. This study sought to identify whether blocking endothelin receptors eliminates the sex-difference in sodium excretion in response to a high salt diet. We treated male and female rats with a blocker for endothelin receptors and evaluated sodium handling by the kidney. Blockade of endothelin receptors increased sodium excretion in male rats fed a high salt diet; whereas sodium excretion in female rats was not affected by blocking endothelin receptors. These data indicate that ET receptors contribute to male–female differences in sodium handling during adjusting to an increased dietary salt.

## Introduction

Sodium (Na^+^) homeostasis is critical for many physiological functions including blood pressure regulation [1], however, the current understanding of Na^+^ handling is based on studies done primarily in males. Our recent studies showed that female Sprague Dawley (SD) rats have a robust natriuretic response after one day of increasing dietary Na^+^ intake, while male rats slowly increase Na^+^ excretion over 3 to 5 days following high salt (HS) diet initiation [2]. This enhanced natriuretic capacity of females during acclimation to an increased dietary salt could be an important protective mechanism that contributes to sex differences in prevalence and progression of hypertension and associated cardiovascular and kidney disease.

Multiple natriuretic pathways contribute to the regulation of Na^+^ homeostasis and consequently blood pressure [3–7]. Renal endothelin-1 (ET-1) signaling plays a critical role in the maintenance of Na^+^ balance via regulating renal tubular reabsorption of Na^+^ [5, 8]. Genetic deletion of ET-1 from the collecting duct in mice led to hypertension and Na^+^ retention [9]. ET-1 downstream signaling is mediated via two G protein-coupled receptors: ET receptor subtype A (ET_A_) and ET receptor subtype B (ET_B_). It has been shown that ET_A_ stimulates vasoconstriction while ET_B_ stimulates vasodilation, natriuresis, and ET-1 clearance [8, 10]. Evidence implicates an important role for renal ET-1 in sex-differences in renal Na^+^ handling [11–14]. Specifically, binding studies revealed that inner medullary collecting duct cells from male SD rats have more ET_A_ than female rats, however no sex-difference in ET_B_ was observed pointing to a higher renal ET_A_ to ET_B_ ratio in males compared to females [14]. In addition, renal medullary ET_A_ contributes to natriuresis in female, but not male, SD rats [15]. Studies utilizing ET_B_ deficient rats demonstrate that ET_A_ plays a dominant role in hypertension development in male rats while ET_B_ and nitric oxide (NO) signaling are protective in female rats [16, 17]. NO and ET-1 have been shown to interact by several direct and indirect mechanisms [18]. One established interaction that may play a role in sex-differences in renal Na^+^ handling is the fact that NO production is stimulated when ET-1 signals through ET_B_ [19].

Recent evidence from our lab points to female SD rats having a more enhanced renal ET-1 system than males [2]. In consistency with these findings in rats, we have also reported that females have higher urinary ET-1 excretion levels in mice and humans [2, 20]. Additionally, one day of dietary Na^+^ loading increases ET_A_ expression in the renal outer medulla of female SD rats while increasing ET_B_ expression in the renal outer medulla of male SD rats [2]. These findings suggest a potential for ET-1 signaling through both receptors ET_A_ and ET_B_ as an important mediator of the sex-difference in Na^+^ excretion observed during acclimation to a HS diet [2]. Studies revealed a sex-difference in ET receptor response following renal ET-1 medullary infusion in normotensive rats [15] as well as angiotensin II hypertensive rats [21]. These data all point to a role for the ET system in sex-differences in salt handling and hypertension.

Despite established differences between males and females in renal ET-1 signaling [11–13], the role of ET receptors in sex-differences in acclimation to dietary Na^+^ challenges is not completely understood. Therefore, the goal of this study was to test the hypothesis that ET receptor function accounts for the augmented natriuretic capacity of female rats, compared to male rats, during acclimation to a HS diet. Given the stark sex-specific differences in natriuresis the upregulation of renal outer medullary ET_A_ and ET_B_ after one day of HS, we evaluated the impact of dual ET_A_ and ET_B_ receptor antagonism on natriuresis in male and female rats maintained on a normal salt (NS) diet and after one day of HS.

## Methods

### Animal studies

Male and female SD rats were purchased from Envigo (Indianapolis, IN). Throughout the study, animals were housed individually in a temperature‐controlled room (22–24 °C) with a 12:12‐h light–dark cycle, with access to food and water ad libitum. All animal protocols were approved by the University of Alabama at Birmingham Institutional Animal Care and Use Committee in accordance with the Guide for the Care and Use of Laboratory Animals.

#### Experimental timeline

**Fig. 1:**
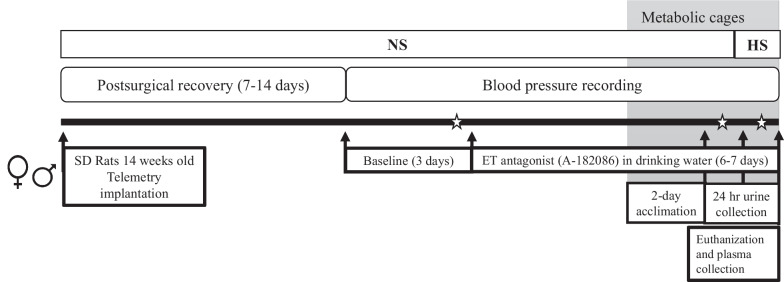
Experimental timeline. Endothelin receptor (ET), high salt (HS, 4% NaCl), Sprague Dawley (SD), and normal salt (NS, 0.3% NaCl). ✩ Designates the blood pressure data displayed in Fig. 4

At 14 weeks of age, male and female rats were implanted with radio telemeters, as detailed below, for continuous blood pressure measurement. Following surgical implantation, rats were allowed to recover for 7 to 14 days before blood pressure recording was initiated. After baseline blood pressure recording, rats were blindly assigned to control or treatment groups. Male and female rats assigned to treatment groups received the dual ET receptor antagonist A-182086 (10 mg/kg/day) [22, 23] in drinking water for a total of 6–7 days (Fig. 1). Male and female rats assigned to control groups received regular drinking water provided by the animal facility. After 2–3 days of initiating A-182086 treatment, rats underwent a metabolic cage study and were switched from a NS diet to a HS diet for 1 day (details below). Then, animals were euthanized between 8 and 10 am and blood was collected for serum through the abdominal aorta (Fig. 1).

#### Drug treatment

A stock of A-182086 (30 mg/100 ml, Abbott Laboratories, Abbott Park, IL), a dual ET receptor antagonist with a balanced affinity for ET_A_ and ET_B_ [24], was prepared in distilled deionized water. The stock was then diluted using drinking water provided by the animal facility to generate a working solution based on the animal’s water intake. Water intake was monitored on daily basis to ensure proper drug dosing throughout the treatment period.

#### Telemetry

Rats were anesthetized with Fluriso (2% isoflurane, 502017, VetOne, Boise, ID) and implanted with HD-S10 transmitters (Data Sciences International, Duluth, MN) as previously described [25] for continuous monitoring of blood pressure by radiotelemetry. Briefly, a midline abdominal incision was made, and the descending aorta was exposed and briefly occluded. The telemeter catheter was inserted into the aorta and secured in place with tissue glue (1469SB, Vetbond, ThermoFisher Scientific, Waltham, MA). The implant was sutured to the abdominal wall and the abdominal incision was closed. After postsurgical recovery, blood pressure recording was initiated, and 3 stable days of baseline blood pressure was collected prior to drug treatment. Recording continued throughout the rest of the study. Mean arterial blood pressure was recorded for 10 s every 10 min interval for the whole experimental timeline.

#### Metabolic cage study and dietary intervention

After 2–3 days on A-182086 treatment, animals were placed into metabolic cages to acclimate for 2 days, then 24-h urine samples were collected while food and water intake were monitored for one day of NS then one day of high salt (HS). 24-h urine sample collection was performed at 8 am. Rats were maintained on a NS diet (0.3% NaCl, NIH-31; Envigo, Indianapolis, IN) for all the experimental timeline except the last day of the study when a nutrient‐matched HS diet (4% NaCl, TD 92034; Envigo, Indianapolis, IN) was introduced for one day before euthanasia.

### Assays

#### Electrolyte measurement

Urine electrolyte concentrations were determined using an EasyLyte analyzer (Medica, Bedford, MA) following the manufacturer’s instructions.

#### ET-1 measurement

Serum and urinary levels of ET-1 were measured using a commercially available QuantiGlo ELISA kit following the manufacturer’s protocol (QET00B, R&D Systems, Minneapolis, MN). The minimum detectable dose of the assay is 0.064 pg/ml. The intra-assay precision CV value is 3.1% while the inter-assay precision CV value is 6.7%.

#### Measurement of NO metabolites

Urinary levels of total NO metabolites (NOx: nitrites and nitrates) were assessed using a commercially available Griess-based NO assay kit following the manufacturer’s protocol (EMSNO, ThermoFisher Scientific, Waltham, MA). The minimum detectable dose of nitrite is 0.222 µM and the minimum detectable dose of nitrate is 0.625 µM. The intra-assay precision CV value for nitrite is 2.4% and for nitrate is 1.5% while the inter-assay precision CV value for nitrite is 7.7% and for nitrate is 3.4%.

### Statistical analysis

GraphPad Prism 9.3.1 was used for figure preparation and statistical analysis. Values are presented as mean ± SEM in all figures and tables. Statistical tests used for each data set are specified in the figure legend. Blood pressure was analyzed via a repeated-measures two-way ANOVA. All other data were evaluated using a two-way ANOVA. *P* values for each data set are displayed in the associated figure or table. *P* < 0.05 was considered significant. Post hoc Sidak’s test was performed following the two-way ANOVA analysis. Significant values from the post hoc analysis are displayed on the graph. D’Agostino–Pearson omnibus normality test was run for all data sets presented. In instances where data failed normality, a Mann–Whitney rank sum test was performed to confirm statistical conclusions.

## Results

### Body weight

**Fig. 2:**
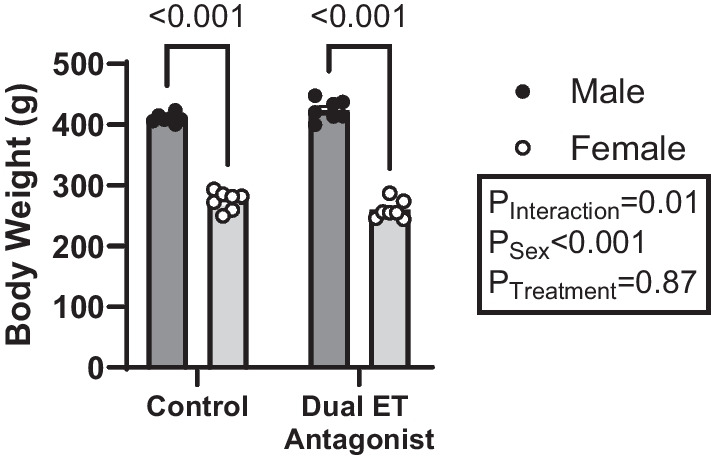
Body weight was not altered by dual ET receptor antagonist treatment in either sex. Male rats weigh more than female rats and dual ET receptor antagonist treatment (A-182086, 10 mg/kg/day) did not alter body weight in either sex. Body weight was measured prior to the metabolic cage experiment. *N* = 7 per group. Two-way ANOVA with post hoc Sidak’s test. *P* values < 0.05 from post hoc test are displayed on graph. Endothelin receptor (ET)

Dual ET receptor antagonist treatment did not alter body weight in either male or female rats (Fig. 2). As expected, male rats weighed more than age-matched female rats, regardless of their treatment (Fig. 2). Given the stark sex-difference in body weight, Na^+^ intake, water intake, urine flow, and urinary excretion data were normalized to body weight to allow for more direct sex comparisons.

### Serum ET-1

**Fig. 3:**
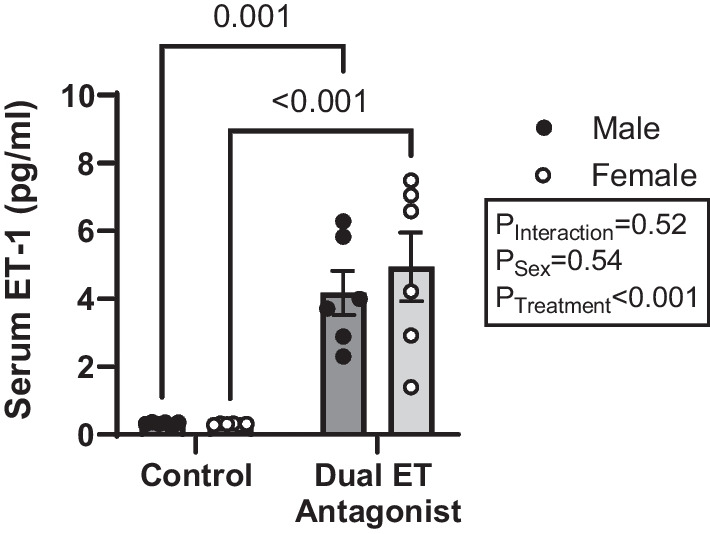
Dual ET receptor antagonist treatment increased serum ET-1 levels in both male and female rats to a comparable extent. Serum levels of ET-1 peptide measured in HS-fed male and female rats treated with or without the dual ET receptor antagonist (A-182086, 10 mg/kg/day). *N* = 6 per group. Two-way ANOVA with post hoc Sidak’s test. *P* values < 0.05 from post hoc test are displayed on graph. Endothelin receptor (ET) and Endothelin-1 (ET-1)

Since ET-1 is cleared through the ET_B_ receptor, efficient ET_B_ receptor blockade is reflected as elevated levels of circulating ET-1 as documented earlier [17, 26]. Thus, serum concentrations of ET-1 were used as a measure of effective drug delivery. No sex-difference was observed in serum ET-1 in control or treated groups. Dual ET receptor antagonism evoked remarkable elevations in serum ET-1 to a similar extent in male and female rats, compared to control rats (Fig. 3).

### Blood pressure

**Fig. 4:**
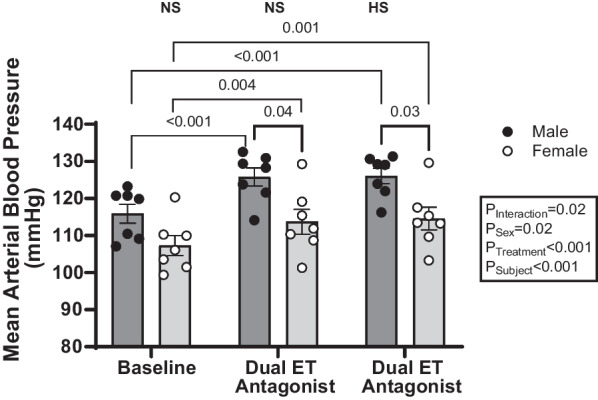
Dual ET receptor antagonism increased blood pressure in both male and female rats. Mean arterial blood pressure measured in NS or HS-fed male and female rats treated with and without the dual ET receptor antagonist (A-182086, 10 mg/kg/day). *N* = 7 per group. Two-way repeated-measures ANOVA with post hoc Sidak’s test. *P* values < 0.05 from post hoc test are displayed on graph. Endothelin receptor (ET), high salt (HS), and normal salt (NS)

Figure 4 depicts 24-h mean arterial blood pressure at baseline and during the days of NS and HS urine collection while animals were treated with A-182086. At baseline, male rats trended to have a higher mean arterial pressure than female rats, though it was not significantly different as assessed via two-way ANOVA. Administration of the dual ET receptor antagonist A-182086 increased mean arterial blood pressure in both male and female rats fed a NS or a HS diet (Fig. 4). No additional alterations in blood pressure in either sex were observed on the first day of increasing the dietary Na^+^ intake (Fig. 4). A-182086-treated male rats on both NS and HS diet had a significantly higher blood pressure than the corresponding female rats (Fig. 4). In response to A-182086 treatment, male rats elicited a greater increase in blood pressure from baseline, compared to female rats while maintained on a NS diet (male vs female; 9.9 ± 0.9 vs 6.4 ± 1.2 mmHg difference between baseline values to values obtained from A-182086-treated rats maintained on a NS *P* = 0.0373). This greater increase in blood pressure in males was also evident on day one of HS.

### Sodium intake and excretion

**Fig. 5:**
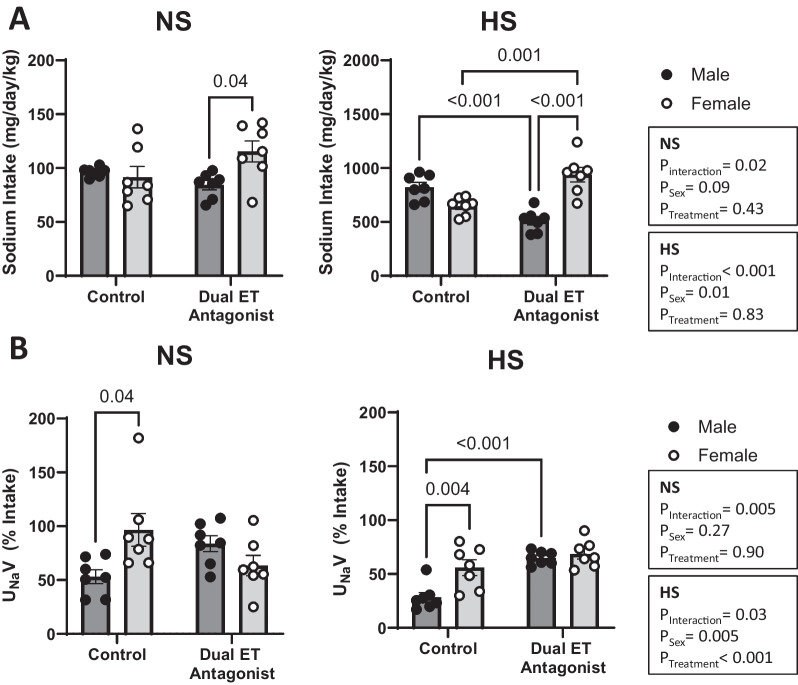
Dual ET receptor antagonism modulated Na^+^ intake and excretion in a sex-dependent manner. Na^+^ intake (**A**) and U_Na_V (**B**) in male and female rats fed a NS or a HS diet. Rats were either treated with or without the dual ET receptor antagonist (A-182086, 10 mg/kg/day). Na^+^ excretion was measured in urine collected over 24 h and expressed as a percent of Na^+^ intake. *N* = 7 per group. Two-way ANOVA with post hoc Sidak’s test. *P* values < 0.05 from post hoc test are displayed on graph. Endothelin receptor (ET), high salt (HS), normal salt (NS), sodium (Na^+^), and urinary sodium excretion (U_Na_V)

On a NS diet, male and female control rats did not elicit differences in food and therefore Na^+^ intake (Table 1, Fig. 5A). Dual ET receptor antagonism uncovered sex-specific differences in food intake. In particular, A-182086-treated female rats consuming a NS diet had higher food, and thus Na^+^, intake compared to corresponding NS-fed A-182086-treated male rats (Table 1, Fig. 5A). On a HS diet, female control rats tended to consume less food and consequently less Na^+^, compared to corresponding male control rats (Table 1, Fig. 5A), however, this sex-difference did not reach statistical significance (*P* = 0.084). Conversely, A-182086-treated male rats maintained on a HS diet consumed less food, and thus less Na^+^, compared to the corresponding HS-fed treated female rats (Table 1, Fig. 5A). This sex-difference in Na^+^ intake in HS-fed treated rats was driven by a decrease in food intake in males and an increase in food intake in females in response to ET receptor antagonism (Table 1, Fig. 5A).

**Table 1:** The effect of dual ET antagonism on metabolic cage parameters in male and female rats fed a normal or high salt diet

Diet	Normal salt (0.3% NaCl)				
Treatment	Control		Dual antagonist		ANOVA results
Sex	Male	Female	Male	Female	
Food intake g/kg/day	50.0 ± 0.84	47.5 ± 5.17	43.7 ± 2.29	59.8 ± 5.07*	*P*_Interaction_ = 0.0224 *P*_Sex_ = 0.0868 *P*_Treatment_ = 0.4324
Water intake ml/kg/day	79.6 ± 5.51	88.3 ± 9.97	66.8 ± 5.29	109.1 ± 11.7*	*P*_Interaction_ = 0.0620 *P*_Sex_ = 0.0068 *P*_Treatment_ = 0.6482
Urine flow ml/kg/day	26.7 ± 2.23	35.4 ± 8.46	25.1 ± 4.77	32.5 ± 8.67	*P*_Interaction_ = 0.8527 *P*_Sex_ = 0.2446 *P*_Treatment_ = 0.6587
U_Na_V mg/kg/day	50.9 ± 5.9	82.4 ± 7.2	69.7 ± 6.2	73.2 ± 14.3	*P*_Interaction_ = 0.1360 *P*_Sex_ = 0.0658 *P*_Treatment_ = 0.6008
U_K_V mg/kg/day	247.0 ± 16.4	296.5 ± 52.4	233.8 ± 14.4	260.5 ± 32.1	*P*_Interaction_ = 0.7302 *P*_Sex_ = 0.2539 *P*_Treatment_ = 0.4579
U_Cl_V mg/kg/day	138.8 ± 11.2	217.0 ± 40.4	163.8 ± 10.6	172.7 ± 25.0	*P*_Interaction_ = 0.1780 *P*_Sex_ = 0.0943 *P*_Treatment_ = 0.7022

Diet	High salt (4% NaCl)				
Treatment	Control		Dual antagonist		ANOVA results
Sex	Male	Female	Male	Female	
Food intake g/kg/day	52.3 ± 2.86	41.1 ± 1.97	32.5 ± 2.45^†^	59.7 ± 4.27^*††^	*P*_Interaction_ < 0.0001 *P*_Sex_ = 0.0138 *P*_Treatment_ = 0.8375
Water intake ml/kg/day	98.1 ± 3.61	129.2 ± 18.20	110.4 ± 11.81	195.1 ± 15.96^*††^	*P*_Interaction_ = 0.0831 *P*_Sex_ < 0.0001 *P*_Treatment_ = 0.0030
Urine flow ml/kg/day	45.4 ± 2.66	71.9 ± 8.94	57.3 ± 6.91	122.1 ± 25.63^*^	*P*_Interaction_ = 0.1913 *P*_Sex_ = 0.0025 *P*_Treatment_ = 0.0337
U_Na_V mg/kg/day	225.6 ± 28.4	368.1 ± 56.5	334.6 ± 31.7	659.5 ± 91.2^*††^	*P*_Interaction_ = 0.1272 *P*_Sex_ = 0.0005 *P*_Treatment_ = 0.0020
U_K_V mg/kg/day	357.0 ± 17.0	366.2 ± 38.3	261.7 ± 17.9	391.0 ± 33.6*	*P*_Interaction_ = 0.0443 *P*_Sex_ = 0.0222 *P*_Treatment_ = 0.2253
U_Cl_V mg/kg/day	410.4 ± 46.3	621.8 ± 87.2	523.8 ± 48.0	1001.9 ± 123.1^*††^	*P*_Interaction_ = 0.1189 *P*_Sex_ = 0.0003 *P*_Treatment_ = 0.0063

*P < 0.05 vs. corresponding male animals within the same treatment group. ^†^*P* < 0.05 vs. corresponding male animals in the control group. ^††^*P* < 0.05 vs. corresponding female animals in the control group. *N* = 7 per group. Two-way ANOVA with post hoc Sidak’s test

Since we observed treatment-specific differences in Na^+^ intake in male and female rats, urinary Na^+^ excretion is presented in Fig. 5B as a percent of Na^+^ intake. On a NS diet, female control rats excreted a greater proportion of dietary Na^+^ than males (Fig. 5B). The urinary Na^+^ excretion data for rats maintained on a NS diet failed normality, so a Mann–Whitney rank sum non-parametric test was performed to confirm the results (control: male vs female *P* = 0.007, dual ET antagonist: male vs female *P* = 0.128). Dual ET receptor antagonism eliminated this difference in part by reducing the percent Na^+^ excreted, albeit not statistically significant, in A-182086-treated female rats compared to control and in part by increasing the percent Na^+^ excreted, albeit not statistically significant, in A-182086-treated male rats compared to control (Fig. 5B). On a HS diet, female control rats excreted a greater fraction of Na^+^ consumed than males (Fig. 5B). Dual ET receptor antagonism eliminates this difference by increasing the apparent natriuretic response in A-182086-treated male rats compared to control (Fig. 5B).

No significant sex-differences in water intake or urine flow were observed by post hoc analysis in control rats maintained on a NS or a HS diet (Table 1). On a NS diet, A-182086-treated female rats had higher water intake, but not urine flow, compared to A-182086-male treated rats (Table 1). In addition, dual ET receptor antagonism to HS-fed animals resulted in greater water intake and urine flow in A-182086-treated female rats compared to A-182086-treated male rats (Table 1). Given the increased water intake in A-182086-treated female rats, we evaluated urine flow as a percent of water intake. However, no sex nor treatment differences in urine flow were observed in HS-fed rats (males: control vs A-182086-treated; 46.5 ± 2.6 vs 52.8 ± 5.6% *P* = 0.887; females: control vs A-182086-treated; 57.2 ± 4.9 vs 59.7 ± 9.5% *P* = 0.992).

On a NS diet, no sex or treatment-related differences in potassium and chloride excretion were observed (Table 1). On a HS diet, A-182086-treated female rats excreted more potassium and chloride than male A-182086-treated rats (Table 1). A-182086-treated female rats also excreted more chloride than control female rats on a HS diet (Table 1).

### Urinary ET-1 and NO excretion

**Fig. 6:**
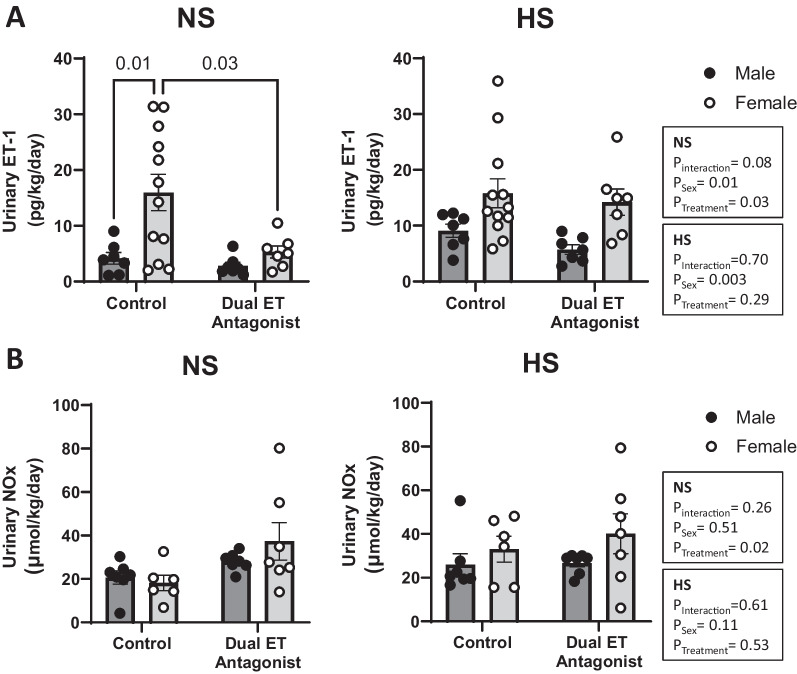
Dual ET receptor antagonism eliminated the sex-difference in urinary ET-1 excretion. Urinary excretion rate of ET-1 (**A**) and NOx (**B**) in male and female rats fed a NS or a HS diet. Rats were either treated with or without the dual ET receptor antagonist (A-182086, 10 mg/kg/day). *N* = 7–13 per group. Two-way ANOVA with post hoc Sidak’s test. *P* values < 0.05 from post hoc test are displayed on graph. Endothelin receptor (ET), endothelin 1 (ET-1), high salt (HS), normal salt (NS), and total nitric oxide metabolites (NOx)

Urinary excretion of ET-1 can be used to reflect intrarenal production levels of this peptide. We found that NS-fed female control rats had greater urinary ET-1 excretion than NS-fed male control rats (Fig. 6A). Dual ET receptor antagonism eliminated the sex-difference in urinary ET-1 excretion (Fig. 6A) by reducing ET-1 peptide excretion rate in female rats (Fig. 6A) while urinary ET-1 excretion in males remained unchanged (Fig. 6A). On a HS diet, there was an overall sex-difference in ET-1 urinary excretion with female rats excreting significantly more than males. Post hoc analysis, however, did not reveal any significant differences between the individual HS-fed groups (Fig. 6A). The female-specific attenuation in ET-1 excretion in response to dual ET antagonism on a NS diet was not evident in animals fed a HS diet. The urinary ET-1 excretion data on a high salt diet failed normality, so Mann–Whitney rank and sum analysis was performed (control: male vs female *P* = 0.0556, dual ET antagonist: male vs female *P* = 0.0041). Given that NO mediates the natriuretic effects of ET-1, we also assessed the urinary levels of NO metabolites (NOx). We did not observe any sex-related differences in the urinary excretion of NOx on either a NS or a HS diet (Fig. 6B). A-182086 treatment resulted in an overall effect on urinary NOx excretion in rats fed a NS, but not a HS, diet. A-182086 overall increased urinary NOx excretion in NS-fed rats, however, post hoc analysis did not show differences in NOx excretion in response to A-182086 treatment in either sex.

## Discussion

Historical understanding of Na^+^ handling, which is critical for blood pressure regulation [1], is based on studies conducted primarily in male subjects. Our lab recently showed that consistent with historical dogma, male rats take 3 to 5 days to increase urine excretion in acclimation to an increased dietary Na^+^ intake, however female rats acclimate on the very first day of HS [2]. The renal ET-1 signaling is an important player in sex-differences in HS-induced natriuretic response as evidence by Ingenuity pathway analysis of RNA sequencing data, urinary ET-1 excretion, as well as renal ET receptor expression changes in response to increasing dietary salt for one day [2]. While several animal and human studies have identified sex and sex hormone-related differences in ET receptor expression and function [8, 11–13, 27–29], the role of ET receptors in sex-differences in acclimation to a HS diet is less clear. The current study revealed that dual ET receptor antagonism eliminates the sex-difference in natriuresis following one day of increased dietary Na^+^ intake by promoting urinary Na^+^ excretion in male rats, rather than attenuating natriuresis in female rats.

Similar to prior studies in humans and rats [2, 30, 31], the present study showed that female rats on a HS diet excrete more Na^+^ as a percent of intake than male rats. Veiras et al. demonstrated that female rats have a more robust natriuretic response to an intraperitoneal saline load than male rats [30]. Similarly, Stachenfeld et al. showed that women elicit a more robust natriuretic response to short-term intravenous infusion of hypertonic saline than men [31]. Furthermore, the current study uncovered an unexpected sex-specific difference in urinary Na^+^ excretion on a NS diet given our observation that female rats excrete more Na^+^, as a percent of intake. Whether male–female differences in Na^+^ gastrointestinal absorption, tissue distribution, and fecal excretion contribute to the observed sex-specific discrepancies in natriuresis remain to be determined.

We hypothesized that ET receptors mediate the augmented natriuretic capacity of female rats, compared to male rats, during acclimation to a HS diet. We anticipated that dual ET receptor antagonism would therefore eliminate the sex-difference in Na^+^ excretion on the first day of HS by attenuating the enhanced natriuretic response observed in females. Dual ET receptor antagonism did eliminate the sex-difference in Na^+^ excretion on day one of HS, however this occurred by increasing Na^+^ excretion in male rats. Specifically, there is a remarkable increase in Na^+^ excretion in HS-fed male rats in response to dual ET receptor antagonism. While we expected the female response to be blunted given the established sex-differences in renal ET_A_ to ET_B_ ratios and the contribution of renal ET_A_ to the regulation of renal vascular tone and Na^+^ excretion [14, 15], the female natriuretic response was unchanged. The increased natriuretic response in males could be related to established male–female differences in ET_A_ to ET_B_ ratios [14] and the potential hemodynamic influence of the ET system, but more work is needed to fully understand the mechanism. Infusion of ET-1 to the renal medulla induces a remarkable decrease in renal medullary blood flow of male, but not female, rats [15]. In addition, renal medullary infusion of ET-1 evokes natriuresis in female rats only, suggesting that ET_A_ activation by ET-1 limits renal medullary blood flow and consequently Na^+^ excretion in male rats [15]. Given the vasoconstrictor contribution of ET_A_ in males, ET-1 may contribute to decreasing renal medullary blood flow and thus limiting natriuresis in un-treated rats. Thus, blockade of ET receptors in males may promote renal blood flow, particularly in the medulla of the kidney, allowing natriuretic pathways to prevail in males while compensatory mechanisms keep the female natriuretic response intact. The current study does not allow us to evaluate the effect of each ET receptor independently and therefore additional studies are needed to better understand the sex-specific effects of each individual receptor on Na^+^ excretion.

Dual ET receptor antagonism resulted in an unexpected increase in food and thus Na^+^ intake in female, but not male rats, on a NS diet. This sex-specific effect is amplified in HS-fed animals as A-182086-treated male rats elicit a reduction in food intake while A-182086-treated female rats increase their food intake. To our knowledge, no existing literature has shown that ET receptors alter food intake or uncover a sex-difference in salt appetite. Interestingly evidence suggests a link between ET signaling and leptin [32, 33] and ghrelin [34, 35], which are two hormones involved in control of appetite and food intake. Parallel to the increase in food/salt intake observed in females, A-182086-treated female rats also increase water intake on both a NS and HS diet. While this increase in food and water intake in A-182086-treated female rats is interesting, its significance and impact on sex-differences in natriuresis pose a new line of inquiry.

The current findings demonstrate that dual ET receptor antagonism results in an increase in blood pressure in both male and female rats. ET_B_ antagonism has previously been shown to increase blood pressure in rats [36, 37] due to an increase in circulating ET-1 as well as changes in ET_A_ expression and this hypertensive response can be attenuated by blocking ET_A_ [36, 38]. Dual ET receptor antagonist effects on blood pressure in experimental animals are variable. A single intravenous injection of A-182086 (12 mg/kg) lowers blood pressure over 60 min in male anesthetized sham and DOCA salt treated rats [39]. In addition, male angiotensin II-hypertensive rats elicit a reduction in blood pressure when treated with A-182086 (24 mg/kg/day) for 5 days [40]. Conversely, treatment of male Dahl salt sensitive rats with A-182086 (30 mg/kg/day) for three days did not alter blood pressure [41]. Clinically, dual ET receptor antagonists have been approved to treat pulmonary arterial hypertension and scleroderma digital ulcers, but have struggled with fluid and salt-retention side effects for other indications largely due to high dosage [42]. It is important to note that male rats elicited a more pronounced increase in blood pressure than female rats, in response to A-182086 treatment. This difference in blood pressure suggests enhanced natriuresis in male rats through pressure natriuresis [3]. This effect on blood pressure poses a limitation to this study. It is important to note this study focused on the first day of acclimation to increased dietary Na^+^ and the interaction between sodium handling and sex difference in blood pressure regulation could be more pronounced in long-term studies. Future studies are needed to understand the mechanisms through which chronic A-182086 treatment is increasing blood pressure in healthy male and female.

ET-1 levels found in the serum and urine can be used to assess ET receptor blocker efficacy and renal production of the peptide, respectively. Since ET-1 is cleared through the ET_B_ receptor, blockade of ET_B_ results in an increase in circulating ET-1 [8, 17, 26, 36]. As expected, both male and female rats treated with the dual ET receptor antagonist had an increase in serum ET-1 confirming effective drug dosing. Urinary ET-1 excretion has been shown to be a reflection of renal ET-1 production [43]. Consistent with previous studies [2, 20], NS-fed female rats excrete greater levels of ET-1 than males indicating a greater intrarenal peptide production. It is important to note that control females have a large variation in urinary ET-1 excretion. Additional experiments are needed to test whether this variation results from the female estrus cycle. This variation in urinary ET-1 excretion in females has been seen in our previous studies [2, 20]. A-182086 treatment eliminates the sex-difference in ET-1 excretion by attenuating urinary ET-1 excretion in females, indicative of a reduction in intrarenal production. Interestingly, dual ET receptor antagonism no longer reduces the ET-1 excretion in female rats fed a HS diet. This observation suggests that salt loading to female rats is likely able to overcome the suppression of ET-1 production observed in response to A-182086 treatment under NS. ET-1 increases NO production through ET_B_ activation [44] and so we also evaluated urinary excretion of NO metabolites. On a NS diet, there was no sex-difference in urinary NOx excretion, however, urinary NOx appeared to increase in female rats in response to dual ET receptor antagonism although this was not significant. ET_B_ receptor activation is not the only source of urinary NO so these results do not provide a clear explanation. Typically, with ET_B_ deficiency or antagonism, NO production and activity are impaired [16, 17, 21]. This increase in urinary NOx is likely ET-1-independent and therefore could be a compensatory mechanism in females that could be exploited in future experiments. On HS diet there are no sex or treatment-related differences in urinary NOx. Urinary NOx does not strictly reflect renal production of NO [45]. NOx excretion is affected by filtered NO metabolites from systemic production and dietary intake, renal tubular reabsorption of NOx, and intrarenal NO production [45]. Therefore, a more thorough investigation of NO would be necessary in future experiments that evaluate the potential mechanism.

The maintenance of Na^+^ homeostasis is under the control of multiple mechanistic pathways in addition to the renal ET-1 signaling system. It is important to consider the potential contribution of the various Na^+^ regulatory pathways to sex-differences in natriuresis during acclimation to dietary salt challenges. Evidence implicates roles for Na^+^ transporters [46, 47], aldosterone signaling [46, 48], and purinergic signaling [49–51] in eliciting sex-differences in renal salt handling and blood pressure regulation. Further, it has been shown that sex hormones and their downstream signaling cascades contribute to the natriuretic capacity of the kidney and consequently hypertension development. Given the evidence for increased salt sensitivity among women, exacerbated by loss of endogenous estrogen seen in menopause [52], more work needs to be done to refine our knowledge about sex and sex hormone-related discrepancies in renal salt handling Sex differences in natriuresis could play a major role in the sex differences in hypertension prevalence. A better understanding of the mechanisms impacting male–female differences in natriuresis could lead to novel, potentially sex specific, anti-hypertensive therapeutics.

In conclusion, our data suggest that the renal ET system contributes to sex-differences in natriuresis on a NS diet and during acclimation to increased dietary Na^+^ intake. In opposition to our original hypothesis, we observed that ET receptor antagonism promotes HS-induced natriuresis in male rats, rather than attenuating the natriuretic capacity of female rats. Additional studies are needed to understand the exact mechanisms underlying enhancement in Na^+^ excretion in response to ET receptor antagonism in males. Overall, a better understanding of the sex-differences in the interplay between ET-1, ET_A,_ ET_B_, and NO is necessary to inform clinical efforts for management of hypertension in both male and female individuals.

## Data Availability

The data that support the findings of this study are available on request from the corresponding author.

## Abbreviations

A-182086: Dual endothelin receptor blocker
ET: Endothelin
ET-1: Endothelin-1
ET_A_: Endothelin receptor A
ET_B_: Endothelin receptor B
HS: High salt
NO: Nitric oxide
NOx: Nitrite plus nitrate
NS: Normal salt
SD: Sprague Dawley
Na^+^: Sodium
